# Optimization of Aqueous Extraction Conditions for Recovery of Phenolic Content and Antioxidant Properties from Macadamia (*Macadamia tetraphylla*) Skin Waste

**DOI:** 10.3390/antiox4040699

**Published:** 2015-11-12

**Authors:** Adriana Dailey, Quan V. Vuong

**Affiliations:** School of Environmental and Life Sciences, University of Newcastle, Ourimbah, NSW 2258, Australia; E-Mail: adriana.dailey@uon.edu.au

**Keywords:** macadamia, waste, optimization, response surface methodology

## Abstract

The macadamia is native to Australia and is now grown commercially around the world. Macadamia skin, known as waste, has been generated abundantly, but this ample source has had limited uses as a byproduct. The aim of this study was to develop optimal aqueous extraction conditions for the recovery of phenolic compounds and antioxidant properties from macadamia skin using Response Surface Methodology (RSM). Water was selected for optimizing the extraction conditions because it is a cheap, safe, and environmentally friendly solvent. The results showed that the RSM models were reliable for the prediction and evaluation of the tested variables. Within the tested ranges, temperature (°C), time (min), and sample-to-solvent ratio (g/100 mL), and their interactions, did not significantly affect phenolic compound (TPC), flavonoid, proanthocyanidin, CUPRAC, and FRAP contents. However, the time and the sample-to-solvent ratio significantly affected DPPH antioxidant activity and the ratio significantly affected ABTS antioxidant capacity. The optimal extraction conditions for the recovery of phenolic compounds and antioxidant properties were predicted and validated at a temperature of 90 °C, a time of 20 min, and a sample-to-solvent ratio of 5 g/100 mL. At these conditions, an extract with TPC of 86 mg GAE/g, flavonoids of 30 mg RUE/g, and proanthocyanidins of 97 mg CAE/g could be prepared with potent antioxidant capacity.

## 1. Introduction

**Figure 1:**
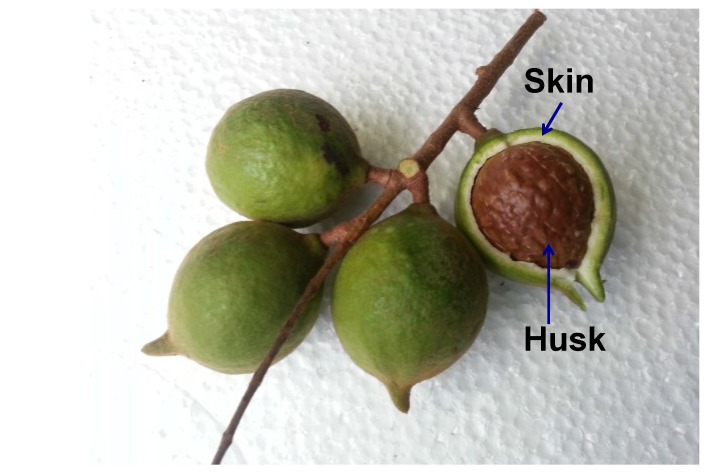
Macadamia (*Macadamia tetraphylla*) nuts.

The macadamia is native to Australia and is now grown commercially around the world [1]. In Australia, there are at least five species of macadamia, but only two species produce edible nuts, including *Macadamia integrifolia* (smooth shelled) and the *Macadamia tetraphylla* (rough shelled), of which the *Macadamia intergrifolia* is the specie most commonly grown for its nuts [2]. Australia is the world’s largest macadamia growing country, providing approximately 28% of the world supply of macadamias to over 40 countries, ahead of South Africa and Hawaii, which supply 25 and 16% of macadamia kernels, respectively. On a yearly basis, Australia produces approximately 40,000 tons of macadamias, with a worth of more than $400 million of economic value to local communities [3]. The macadamia nut itself is mainly sold as the kernel, which only accounts for approximately 20% of the total weight of the nut. The remaining 80% of the nut, including the skin (42%) and the husk (38%) (Figure 1), are often treated as waste products with little value. It is estimated that approximately 16,800 tons of skin and 15,200 tons of husk by-product are generated annually in Australia alone [4]. Several studies have attempted to utilize macadamia by-products, such as the husk being used as a source of fuel filler in the plastic industry [5], the husk and skin as furniture materials [4], and husks as carbon composites for post combustion CO_2_ capture [2]; however, they are far under-utilized, so this abundant source is a great starting material for bioactive compound extraction.

Polyphenols are major bioactive components in fruits, vegetables, cereals, oilseeds, and nuts; approximately 8000 individual compounds have been identified [6]. Due to their widespread occurrence in food materials, they are an integral part of human nutrition. Furthermore, they possess potent antioxidant properties and have been linked to different health benefits. Through their inherent redox properties they can neutralize free radicals and scavenge reactive oxygen species (ROS) [7]. Plant phenolic compounds have been considered as one of the most important and comprehensively incorporated classes of bioactive compounds for cancer prevention and chemotherapy [8]. They have been found to associate with the prevention and reduction of various types of cancer cells *in vitro* and tumors *in vivo* [8]. Phenolic compounds have been reported to reduce the risks of cardiovascular disease (CVD), as well as reducing the incidence of coronary heart disease [9]. There has been limited research on the types and quantities of natural antioxidants within nuts, in particular macadamia nuts, but it has been suggested that phenolic compounds possess a much higher antioxidant capacity than nutrient antioxidants such as Vitamin E and selenium [10]. A large number of studies have acknowledged that the skins of tree nuts are a richer source of phenolic compounds and possess much stronger antioxidant activities than the kernel and other byproducts of nut manufacture [11].

Although macadamia byproducts are abundant and might be a rich source of phenolic compounds; very limited work has been conducted on the use of macadamia byproducts for the recovery of phenolic compounds for further utilization in the food and pharmaceutical industries. Therefore, it is important to optimize the extraction conditions for the recovery of phenolic compounds and antioxidant capacity from macadamia skin. Response surface methodology (RSM) is a useful program that uses an efficacy mathematical and statistical technique for the analysis of empirical models that help to describe the impact of independent variables, such as solvents, extraction temperature, and time, as well as solvent-to-sample ratio, and their interfaces on response variables. The major advantage of using this program is the reduction in the number of experiments that need to be run, resulting in a reduction in time, reagents, and materials [12]. Therefore, the aim of this study was to develop optimal aqueous extraction conditions for the recovery of phenolic compounds and antioxidant properties from macadamia skin using RSM.

## 2. Experimental Section

### 2.1. Materials

Macadamia (*Macadamia tetraphylla*) nuts (Figure 1) were collected in July 2014 in the Central Coast region, New South Wales, Australia (latitude of 33.4°S, longitude of 151.4°E). Once harvested, the skins of the fruits were removed and then immediately frozen in liquid nitrogen and freeze-dried (FD3 freeze dyer, Thomas Australia Pty. Ltd., Seven Hills, NSW, Australia) to minimize oxidation or degradation of bioactive compounds. The dried skin was then ground using a commercial blender (John Morris Scientific, Chatswood, NSW, Australia) and sieved using a steel mesh sieve (1.4 mm EFL 2000; Endecotts Ltd., London, England). The dried, ground skin was kept in a sealed and labelled container at 5 °C for further analysis.

### 2.2. Chemicals

All chemicals used in this study were analytical grade. Acetonitrile, ethanol, methanol, ethyl acetate, vanillin, sulfuric acid, and potassium persulfate were purchased from Merck (Germany). Folin–Ciocalteu phenol regent, anhydrous sodium carbonate, sodium nitrile, ferric chloride, gallic acid, rutin, catechin, neocuproine, 2,4,6-tris(2-pyridyl)-s-triazine, (±)-6-hydroxy-2,5,7,8-tetramethylchromane-2-carboxylic acid (trolox), and 2,2-Diphenyl-1-picrylhydrazyl (DPPH) were purchased from Sigma-Aldrich Co. (New South Wales, Australia). Sodium acetate trihydrate was purchased from Government Stores Department (Australia). Aluminum chloride was obtained from J. T. Baker Chem. Co. (Belgium). Acetic acid was obtained from BDH Laboratory Supplies (Poole, England). Sodium hydroxide was purchased from Ajax Chem. (Australia) and hydrochloride acid was obtained from Lab-scan Ltd. (Ireland). 

### 2.3. Extraction Process 

A water bath (Ratek Instruments Pty. Ltd., Victoria, Australia) was used for the extraction process. Water was used as the solvent system as it is a safe and inexpensive solvent and is easily accessible in comparison with other organic solvents [13]. Parameter combinations were designed by the response surface methodology (RSM) software and the extractions were conducted under these designed conditions (shown in Table 1). The extraction was undertaken in sealed vessels to minimize evaporation. After extraction was completed, the extracts were placed in an ice bath immediately, cooled to room temperature, filtered using filter paper, and diluted up to 160 times for quantitative analysis and antioxidant determination. 

### 2.4. Response Surface Methodology (RSM)

Response Surface Methodology experimental design and analysis were conducted using JMP software (Version 11). A three-level, three-factor, Box–Behnken design was used with three central points replicated in the design of the experimental conditions, based upon preliminary single-factor tests [14]. This exposes the influence of the three significant independent parameters: time (10–30 min), temperature (60–90 °C), and sample-to-solvent ratio (2–5 g/100 mL). The software is also used to establish the model equation, to graph the 3-D plot and 2-D contour of the response, and to predict the optimum values for the three response variables.

To express the level of phenolic compounds and antioxidant properties as a function of the independent variables, a second-order polynomial equation was used as follows [13]:

(1) $$Y\text{​​​​​​​ }=\text{ ​​​​​​​​ ​​}\beta_{o}+\sum_{i=1}^{k}\beta_{i}{Χ}_{i}+\sum_{\begin{array}{l} i=1\\ i<j \end{array}}^{k-1}\sum_{j=2}^{k}\beta_{ij}{Χ}_{i}{Χ}_{j}+\sum_{i=1}^{k}\beta_{ii}{Χ}_{i}^{2}$$

where various *X_i_* values are independent variables affecting the responses *Y*; *β*_0_, *β_i_*, *β_ii_*, *and β_ij_* are the regression coefficients for intercept, linear, quadratic, and interaction terms, respectively; and *k* is the number of variables.

The three independent variables were assigned as *X*_1_ (temperature, °C), *X*_2_ (time, min), and *X*_3_ (ratio, sample-to-water, g/100 mL). Thus, the function containing these three independent variables is expressed as follows:

(2) $$Y=\;\beta_{0}+\beta_{1}X_{1}+\beta_{2}X_{2}+\beta_{3}X_{3}+\beta_{12}X_{1}X_{2}+\beta_{13}X_{1}X_{3}+\beta_{23}X_{2}X_{3}+\beta_{11}X_{1}^{2}+\beta_{22}X_{2}^{2}+\beta_{33}X_{3\;}^{2}.$$

**Table 1:** Box–Behnken design and the observed responses.

Run	Experimental Conditions			Experimental Results						
	X_1_	X_2_	X_3_	TPC	Flavon-Oids	Proantho-Cyanidins	ABTS	DPPH	CUPRAC	FRAP
1	75	10	2	47.19	25.90	37.21	237.50	151.70	465.46	330.73
2	60	20	2	114.27	12.54	113.17	410.70	269.62	407.81	174.42
3	90	20	2	175.27	27.68	173.97	318.80	215.66	606.74	471.53
4	75	30	2	61.14	11.86	53.91	320.82	217.22	263.83	249.65
5	60	10	5	74.64	13.54	62.22	143.57	104.43	116.91	417.68
6	90	10	5	90.92	27.77	93.85	155.14	96.23	167.16	298.43
7	75	20	5	53.57	16.29	51.27	122.08	61.20	265.45	189.69
8	75	20	5	53.82	17.73	54.80	84.37	47.93	217.87	296.44
9	75	20	5	37.73	17.16	34.01	108.56	66.30	182.82	222.23
10	60	30	5	40.94	20.45	34.73	248.49	188.23	438.67	295.28
11	90	30	5	39.21	25.37	38.16	197.63	134.50	247.66	370.50
12	75	10	8	48.08	15.50	40.35	220.56	124.54	193.37	191.98
13	60	20	8	32.59	12.27	34.02	167.26	103.16	103.34	162.78
14	90	20	8	74.99	22.37	71.96	226.84	177.25	282.00	316.90
15	75	30	8	31.32	20.13	32.67	259.22	160.77	456.88	304.03

X_1_ (temperature, °C), X_2_ (time, min) and X_3_ (ratio, water-to-sample, mL/g), TPC (mg GAE/g of dried weight), Flavonoids (mg RUE/g of dried weight), Proanthocyanidins (mg CE/g of dried weight), ABTS (µM TE/g of dried weight), DPPH (µM TE/g of dried weight), CUPRAC (µM TE/g of dried weight), and FRAP (µM·TE/g of dried weight).

### 2.5. Methods for Determination of Chemical Properties

#### 2.5.1. Total Phenolic Content (TPC)

TPC was determined as described by Vuong *et al.* [15]. To 1 mL of diluted sample, 5 mL of 10% (*v/v*) Folin–Ciocalteu reagent was added, followed by the addition of 4 mL of 7.5% (*w/v*) Na_2_CO_3_; the result was then mixed well on a vortex agitator and incubated in the dark at room temperature for one hour before the absorbance was measured at 760 nm using a UV spectrophotometer (Varian Australia Pty. Ltd., Victoria, Australia). Gallic acid was used as the standard for a calibration curve and the results were expressed as milligrams of gallic acid equivalents per gram of sample (mg GAE/g).

#### 2.5.2. Total Flavonoids

The total flavonoid content was measured as described by Zhishen *et al.* [16]. To 0.5 mL of diluted sample, 2 mL of H_2_O and 0.15 mL of 5% (*w/v*) NaNO_2_ were added and left at room temperature for 6 min. Then 0.15 mL of 10% (*w/v*) AlCl_3_ was added and left at room temperature for 6 min. This was followed by the addition of 2 mL 4% (*w/v*) NaOH and 0.7 mL of H_2_O, with the final solution being mixed well and left at room temperature for a further 15 min before the absorbance was measured at 510 nm using a UV spectrophotometer. Rutin was used as the standard for a calibration curve and the results were expressed as milligrams of rutin equivalents per gram of sample (mg RUE/g).

#### 2.5.3. Proanthocyanidins

The content of proanthocyanidins was determined as described by Li *et al.* [17]. To 0.5 mL of diluted sample, 3 mL of 4% (*w/v*) of vanillin was added. The mixture was then added to 1.5 mL of concentrated HCl and left at room temperature for 15 min before measurement of the absorbance at 500 nm using a UV spectrophotometer. Catechin was used as the standard for a calibration curve and the results were expressed as milligrams of catechin equivalents per gram of sample (mg CE/g).

### 2.6. Methods for Determination of Antioxidant Properties

#### 2.6.1. ABTS Radical Scavenging Capacity

ABTS radical scavenging activity was determined according the methods described by Thaipong *et al.* [18] and Kamonwannasit *et al.* [19] with a few modifications. A stock solution was prepared by adding 10 mL of 7.4 mM ABTS solution to 10 mL of 2.6 mM K_2_S_2_O_8_ and left at room temperature in the dark for 15 h, then stored at −20 °C until required. The working solution was freshly prepared by diluting 1 mL of stock solution with 60 mL of methanol to obtain an absorbance value of 1.1 ± 0.02 at 734 nm. To 0.15 mL of sample, 2.85 mL of the working solution was added and mixed, then left in the dark at room temperature for 2 hours before its absorbance was measured at 734 nm using a UV-VIS spectrophometer (Cary 50 Bio, Varian Australia Pty. Ltd., Australia). Trolox was used as a standard and the results were expressed as micromoles of trolox equivalents per gram of dried sample (μM TE/g).

#### 2.6.2. DPPH Radical Scavenging Activity

The radical scavenging activity was measured based on the method described by Thaipong *et al* [18], with some modifications. A stock solution was prepared by dissolving 24 mg DPPH with 100 mL methanol and then stored at −20 °C until required. The working solution was then prepared fresh by mixing 10 mL stock solution with 45 mL methanol to obtain an absorbance at 515 nm of 1.1 ± 0.02. To 0.15 mL of sample, 2.85 mL of working solution was added and then left in the dark at room temperature for 3 hours before measuring the absorbance at 515 nm using the UV spectrophotometer. Trolox was used as the standard for a calibration curve and the results were expressed as micromoles of trolox equivalents per gram of sample (μM TE/g).

#### 2.6.3. Cupric Reducing Antioxidant Capacity (CUPRAC)

CUPRAC was determined as described by Apak *et al.* [20] with some modifications. To 1 mL of CuCl_2_, 1 mL of neocuproine and 1 mL of NH_4_Ac were added, followed by the addition of 1.1 mL of sample. After mixing well, the mixture was incubated at room temperature for 1.5 hours before measuring the absorbance at 450 nm using the UV spectrophotometer. Trolox was used as the standard for a calibration curve and the results were expressed as micromoles of trolox equivalents per gram of sample (μM TE/g).

#### 2.6.4. Ferric Reducing Antioxidant Power (FRAP)

FRAP was measured as described by Thaipong *et al.* [18] and Kamonwannasit *et al.* [19]. A working FRAP solution was prepared by mixing 300 mM acetate buffer, 10 mM TPTZ in 40 mM HCl and 20 mM FeCl3 in the ratio of 10:1:1 and warmed at 37 °C in a water bath (Ratek Instruments Pty. Ltd., Victoria, Australia) before using. To 0.15 mL of sample was added 2.85 mL of the working FRAP solution and incubated at room temperature in the dark for 30 min before its absorbance was read at 593 nm. Trolox was used as a standard and the results were expressed as micromoles of trolox equivalents per gram of dried sample (μM TE/g).

### 2.7. Statistical Analyses

The student’s T-test was conducted using the JMP statistical software (Version 11, SAS Institute Inc., Cary, NC, USA) for comparison of sample means. All experiments were performed in at least triplicate and the results averaged. One-way ANOVA tests are also used for mean comparison between different treatments. Differences between the mean in the different treatments are taken to be statistically significant at *p* < 0.05. 

## 3. Results and Discussion

### 3.1. Statistical Analysis and the Model Fitting

**Figure 2:**
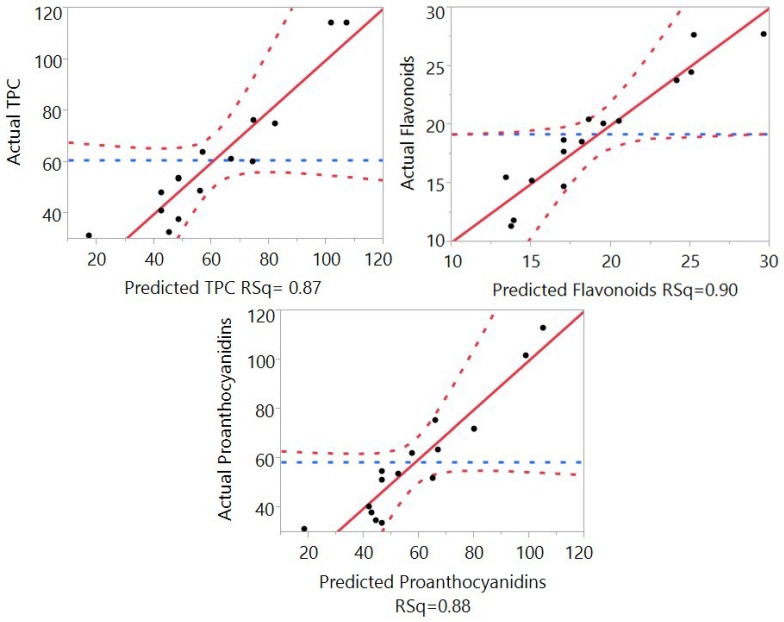
Correlation between the predicted and experimental values for TPC, flavonoids, and proanthocyanidins.

**Figure 3:**
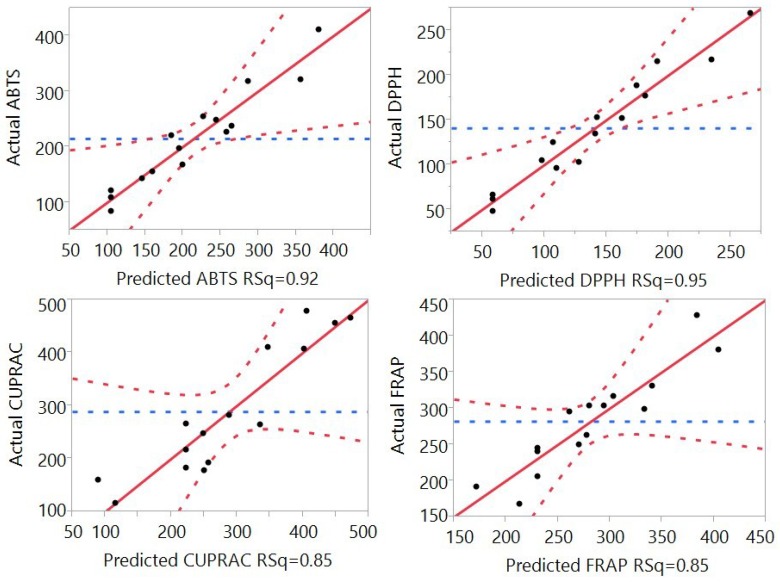
Correlation between the predicted and the experimental values for ABTS total antioxidant capacity, DPPH free radical scavenging capacity, cupric reducing antioxidant power (CUPRAC), and ferric reducing antioxidant power (FRAP).

Fitting the models is critical for interpretation of the accuracy of the RSM mathematical models for prediction of the TPC, flavonoids, proanthocyanidins, and antioxidant capacity from the *Macadamia tetraphylla* skin. The analysis of variances of the Box–Behnken design for the determination of the model fitting is outlined in Table 2 and Figure 2 and Figure 3.

**Table 2:** Analysis of variance for the determination of model fitting.

	TPC	Flavon-Oids	Proantho-Cyanidins	Antioxidant Capacity			
				ABTS	DPPH	CUPRAC	FRAP
Lack of fit	0.21	0.04	0.36	0.12	0.09	0.15	0.14
*R*²	0.87	0.93	0.88	0.92	0.95	0.85	0.85
Adjusted *R*²	0.64	0.80	0.66	0.77	0.86	0.57	0.59
PRESS	16310	487	12077	131264	41294	469190	144892
F ratio of Model	3.81	7.36	4.13	6.48	11.11	3.08	3.25
*P* of model > F	0.07	0.02	0.06	0.03	0.01	0.11	0.10

Fitting of the models for TPC, flavonoids, and proanthocyanidins was analyzed and results are shown in Table 2 and Figure 2. For the total phenolic compounds, the coefficient of determination (*R*^2^) of the model was 0.87, indicating an 87% match between the values of the predicted model and the values that were attained in the experimental data. The p-value for the lack of fit was identified as 0.21, highlighting that the lack of fit of the model was significant at *p* > 0.05. The predicted residual sum of squares (PRESS) value (which is used to compare the predictive power of multiple models) was identified as 16,310. Furthermore, the F value of the model was 3.81, indicating the competency of the model in its ability to predict the extraction yields of TPC. A *p*-value substandard to 0.05 or 0.01 gives the significance of the model, taking into account a confidence interval of 95% or 99% respectively [21]. The *p*-value of the model was 0.07, indicating that it was not deemed significant.

In the case of flavonoids, the results showed that the *R*^2^ value of the model was 0.93 (Figure 2), showing a 93% data match. For the *p*-value for lack of fit, PRESS, *F* value, and *p*-value the data showed 0.04, 487, 7.36, and 0.02 respectively, indicating that the model was significant (*p* < 0.05) and reliable for the prediction of flavonoids. For the predictability of proanthocyanidins in the extraction process, the model found an *R*^2^ value of 0.88, indicating an 88% correlation between the predicted and experimental values (Figure 2). The *p*-value for lack of fit was standing at a significant 0.35, with a PRESS value of 12077, *F* value of 4.13, and a non-significant 0.06 for the *p*-value for the model (*p* > 0.05), revealing that the model is accurate for the prediction of proanthocyanidins. 

Through the application of multiple regression analysis on the experimental data, a projected response Y for the TPC, flavonoids, and proanthocyanidins from *Macadamia tetraphylla* skin can be exhibited through the below second-order polynomial (Equations (3)–(5)):

(3) $$Y_{TPC}=48.37958+7.809985X_{1}-8.28521X_{2}-20.3416X_{3}-1.13514X_{1}X_{2}+10.61487X_{1}X_{3}-4.45646X_{2}X_{3}+21.48476{X_{1}}^{2}-12.3749{X_{2}}^{2}+14.15593{X_{3}}^{2}$$

(4) $$Y_{Flavonoids}=17.06533+4.249875X_{1}-1.26013X_{2}-1.50417X_{3}-1.474X_{1}X_{2}-0.85292X_{1}X_{3}+4.320417X_{2}X_{3}+3.108417{X_{1}}^{2}+2.46425{X_{2}}^{2}-1.52917{X_{3}}^{2}$$

(5) $$Y_{Proanthocyanidins}=\;46.69474+1.725X_{1}-8.99287X_{2}-14.2209X_{3}-2.45439X_{1}X_{2}+4.881579X_{1}X_{3}-2.74233X_{2}X_{3}+24.62555{X_{1}}^{2}-18.6694{X_{2}}^{2}+16.36393{X_{3}}^{2\;}.$$

Fitting of the models for four different antioxidant properties including total antioxidant capacity (ABTS), DPPH free radical scavenging capacity, cupric reducing antioxidant capacity (CUPRAC), and ferric antioxidant power (FRAP) were also analyzed and the results are shown in Table 2 and Figure 3. The results revealed that the coefficient of determination (*R*^2^) for the ABTS, DPPH, CUPRAC, and FRAP models (Table 2) was determined to be 0.92, 0.95, 0.85, and 0.85, respectively, revealing a close correlation between the predicted values and experimental values, with at least 85% of data matching.

PRESS values for the ABTS, DPPH, CUPRAC, and FRAP models (Table 2) were found to be 131264, 41294, 469190 and 469190, respectively. Furthermore, F values for the ABTS, DPPH, CUPRAC, and FRAP models were 6.48, 11.11, 3.08, and 3.25; and the lack of fit was 0.12, 0.09, 0.15, and 0.14, highlighting that the lack of fit of the model was significant at *p* > 0.05. These results revealed that mathematical models were reliable predictors of antioxidant capacity and could be fitted to the following second-order polynomial (Equations (6)–(9)):

(6) $$Y_{ABTS}=105.0044-8.95139X_{1}+33.67389X_{2}-52.2514X_{3}-15.6089X_{1}X_{2}+37.86944X_{1}X_{3}-12.1889X_{2}X_{3}+51.80306{X_{1}}^{2}+29.40583{X_{2}}^{2}+124.0953{X_{3}}^{2}$$

(7) $$Y_{DPPH}=58.47778-5.22604X_{1}+27.03611X_{2}-37.0017X_{3}-11.3833X_{1}X_{2}+32.01042X_{1}X_{3}-9.20139X_{2}X_{3}+51.05938{X_{1}}^{2}+21.31285{X_{2}}^{2}+81.88993{X_{3}}^{2}\;$$

(8) $$Y_{CUPRAC}=222.0527+10.75407X_{1}+55.64071X_{2}-67.3052X_{3}-59.8078X_{1}+8.335271X_{1}X_{3}+124.7403X_{2}X_{3}+10.0905{X_{1}}^{2}+9.952907{X_{2}}^{2}+104.4285{X_{3}}^{2}$$

(9) $$Y_{FRAP}=230.6576+49.23343X_{1}+13.09773X_{2}-36.3954X_{3}+22.71136X_{1}X_{2}-4.04451X_{1}X_{3}+48.2822X_{2}X_{3}+57.01089{X_{1}}^{2}+31.90199{X_{2}}^{2}+6.542708{X_{3}}^{2}.$$

### 3.2. Effect of Extraction Independent Variables on TPC, Flavonoids, and Proanthocyanidins

**Figure 4:**
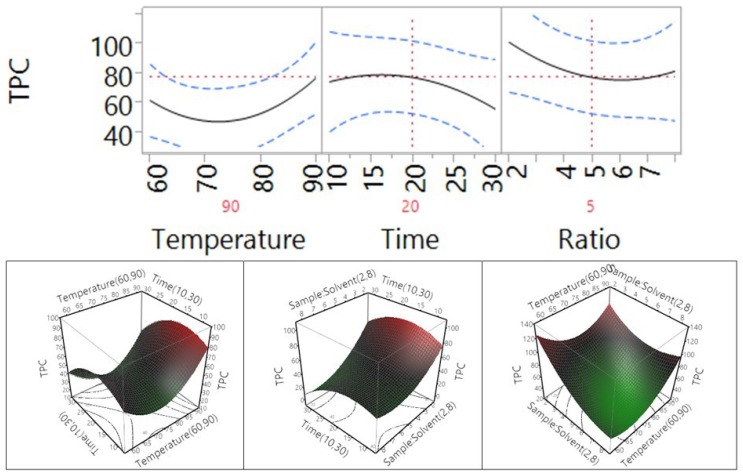
Impact of temperature (60–90 °C), time (10–30 min), and sample-to-solvent ratio (2–8 g/100 mL) on TPC (mg GAE/g).

Effect of temperature, extraction time, and ratio on TPC was shown in Figure 4 and Table 3. The results showed that when the temperature increased from 60 °C to 90 °C, the extraction efficiency of TPC did not increase significantly (*p* > 0.05). When extraction time increased from 10 min to 30 min, the extraction efficiency of TPC did not change significantly. Similarly, when the sample-to-solvent ratio increased from 2 to 8 g/100 mL, the extraction efficiency of TPC also did not vary significantly. Overall, the results indicated that within the narrow range of conditions tested in this study, the extraction efficiency of TPC did not change significantly.

**Table 3:** Analysis of variance for the experimental results on TPC, flavonoids, and proanthocyanidins.

Parameter	DF	TPC		Flavonoids		Proanthocyanidins	
		Estimate	Prob > |*t*|	Estimate	Prob > |*t*|	Estimate	Prob > |*t*|
β_0_	1	48.37	0.0445 *	17.06	0.001 *	46.69	0.05
β_1_	1	14.74	0.2419	3.78	0.07	16.72	0.20
β_2_	1	−11.02	0.3665	−0.57	0.74	−9.2	0.45
β_3_	1	−26.36	0.0637	−1.71	0.35	−24.90	0.07
β_12_	1	−4.50	0.7859	−1.19	0.64	−7.04	0.67
β_13_	1	−4.649	0.7792	−1.73	0.50	−5.71	0.735
β_23_	1	−7.67	0.6457	3.87	0.16	−6.09	0.71
β_11_	1	32.69	0.102	3.18	0.26	33.89	0.09
β_22_	1	−19.64	0.2834	2.11	0.43	−23.34	0.22
β_33_	1	18.20	0.3162	−0.72	0.78	17.69	0.33

* Significantly different at *p* < 0.05; β_0_: Intercept; β_1_, β_2_, and β_3_: Linear regression coefficients for temperature, time and ratio; β_12_, β_13_, and β_23_: Regression coefficients for interaction between temperature × time, temperature × ratio and time × ratio; β_11_, β_22_, and β_33_: Quadratic regression coefficients for temperature × temperature, time × time, and ratio × ratio.

### 3.3. Effect of Extraction-Independent Variables on Flavonoids of Macadamia tetraphylla Skin Extracts

**Figure 5:**
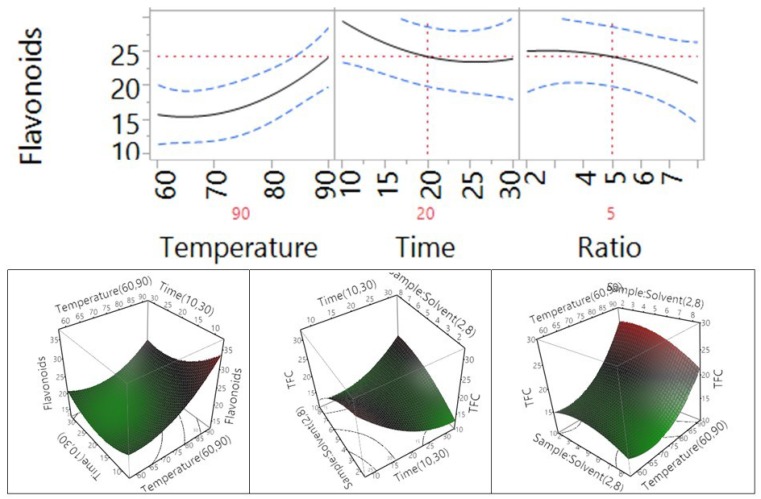
Impact of temperature (60–90 °C), time (10–30 min), and sample-to-solvent ratio (2–8 g/100 mL) on flavonoids (mg RUE/g).

The effects of temperature, extraction time, and ratio on extraction efficiency of flavonoids are shown in Table 3 and Figure 5. The results revealed that all extraction parameters in the tested range in the current study did not significantly affect extraction efficiency of total flavonoids (*p* > 0.05). However, the trends of the changes in extraction efficiency of flavonoids were different. When the temperature increased from 60 °C to 90 °C, the extraction efficiency of flavonoids increased, whereas an opposite trend in the extraction efficiency of flavonoids was observed when the time and the sample-to-solvent ratio increased. In addition, the interactions between the temperature and time, temperature and ratio, and time and ratio had no significant impact on the extraction efficiency of flavonoids (*p* > 0.05, Table 3).

**Figure 6:**
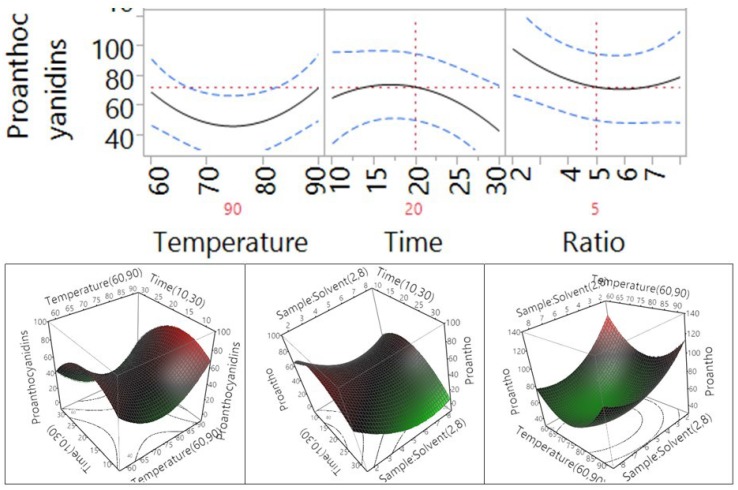
Impact of temperature (60*–*90 °C), time (10–30 min), and sample-to-solvent ratio (2–8 g/100 mL) on proanthocyanidins (mg CAE/g).

The effects of temperature, extraction time, and ratio on extraction efficiency of proanthocyanidins re shown in Table 3 and Figure 6. Similar to TPC and flavonoids, the results indicated that temperature, extraction time, and sample-to-water ratio in the tested range did not significantly affect extraction efficiency of proanthocyanidins (*p* > 0.05). The trends of the changes in extraction efficiency of proanthocyanidins were also similar to the changes of flavonoids. When the temperature increased from 60 °C to 90 °C, the extraction efficiency of flavonoids increased, whereas when the time and the sample-to-solvent ratio increased from 10 to 30 min and from 2 to 8 g/100 mL, respectively, the extraction efficiency of proanthocyanidins decreased.

### 3.4. Effect of Extraction-Independent Variables on the Antioxidant Capacity of Macadamia tetraphylla Skin

The impact of different extraction conditions on antioxidant properties were tested using four antioxidant assays including ABTS, DPPH, CUPRAC, and FRAP. Four antioxidant assays were conducted in this study because there was more confidence of data revealing the antioxidant capacity of *Macadamia tetraphylla,* as each antioxidant assay has advantages and limitations [22]. For example, the ABTS assay can be used over a wide pH range, with different types of solvents. On the other hand, many antioxidants that react quickly with peroxyl radicals may react slowly or may even be insensitive to DPPH due to steric inaccessibility [23]. The FRAP assay actually only measures the reducing capability based upon the ferric ion, which is not relevant to antioxidant activity mechanistically and physiologically [23].

**Figure 7:**
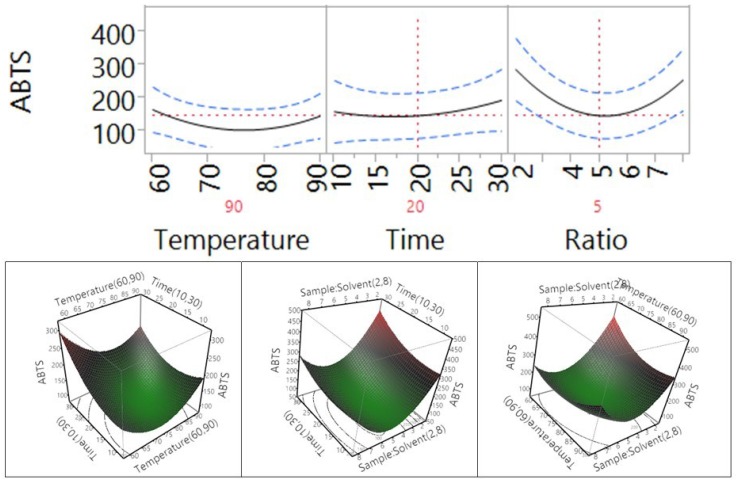
Impact of temperature (60–90 °C), time (10–30 min), and sample-to-solvent ratio (2–8 g/100 mL) on ABTS.

The influence of conventional extraction parameters on the antioxidant ability of *Macadamia tetraphylla* skin using ABTS assay is shown in Table 4 and Figure 7. The results show that temperature and extraction time did not significantly affect the antioxidant properties of the macadamia skin, but the sample-to-water ratio in the tested range significantly affected its antioxidant capacity (*p* < 0.05). When the temperature increased from 60 °C to 90 °C, the trend of antioxidant activity decreased, whereas when the time increased from 10 min to 30 min, the antioxidant capacity increased. Of note, when the sample-to-solvent ratio increased from 1 to 5 g/100 mL, the antioxidant activity significantly decreased and then increased when the ratio further increased from 5 to 8 g/100 mL.

**Table 4:** Analysis of variance for the experimental results on antioxidant capacity.

Parameter	DF	ABTS		DPPH		CUPRAC		FRAP	
		Estimate	Prob > | *t*|	Estimate	Prob > | *t*|	Estimate	Prob > | *t*|	Estimate	Prob > | *t*|
β_0_	1	105.0044	0.0079 *	58.47778	0.0085 *	222.0527	0.0347 *	236.1242	0.0055 *
β_1_	1	−8.95139	0.578	−5.22604	0.5669	29.6031	0.5585	50.89991	0.161
β_2_	1	33.67389	0.0755	27.97708	0.022*	58.01841	0.2742	−2.42235	0.9406
β_3_	1	−51.7403	0.0185 *	−36.0608	0.0083 *	−88.532	0.1199	−31.3291	0.3578
β_12_	1	−15.6089	0.4963	−11.3833	0.3887	−60.3147	0.4082	48.61667	0.3172
β_13_	1	37.86944	0.1354	32.01042	0.0453	−5.06977	0.9425	−35.7491	0.4512
β_23_	1	−11.1667	0.6223	−7.31944	0.5706	116.2849	0.1424	48.2822	0.3202
β_11_	1	51.29194	0.0685	50.1184	0.0104 *	12.8188	0.861	60.82964	0.2393
β_22_	1	29.91694	0.2348	22.25382	0.1366	7.731589	0.9158	48.52188	0.3355
β_33_	1	124.6064	0.0025 *	82.8309	0.0012 *	115.1052	0.1589	−15.5438	0.7468

* Significantly different at *p* < 0.05; β₀: Intercept; β_1_, β_2_, and β_3_: Linear regression coefficients for temperature, time and ratio; β_12_, β_13_, and β_23_: Regression coefficients for interaction between temperature × time, temperature × ratio and time x ratio; β_11_, β_22_, and β_33_: Quadratic regression coefficients for temperature x temperature, time × time, and ratio × ratio.

**Figure 8:**
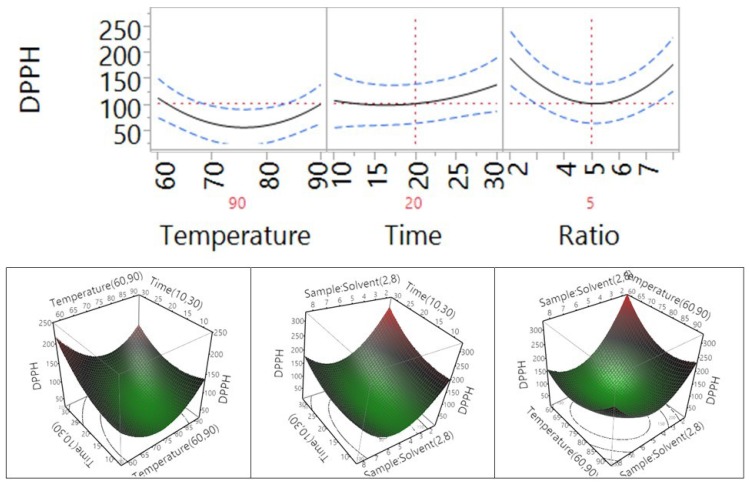
Impact of temperature (60–90 °C), time (10–30 min), and sample-to-solvent ratio (2–8 g/100 mL) on DPPH free radical scavenging capacity.

The impact of temperature, time, and sample-to-solvent ratio on the antioxidant ability of *Macadamia tetraphylla* skin using DPPH assay is shown in Table 4 and Figure 8. The results showed that temperature did not significantly affect the DPPH antioxidant properties of the macadamia skin, but the extraction time and the sample-to-water ratio in the tested range did (*p* < 0.05). When the temperature increased from 60 °C to 75 °C, the trend of antioxidant activity decreased, then increased from that point on until the temperature reached 90 °C. However, when the time increased from 10 min to 30 min, the antioxidant capacity significantly increased. When the sample-to-solvent ratio increased from 1 to 5 g/100 mL, the antioxidant activity significantly decreased and then increased when the ratio further increased from 5 to 8 g/100 mL. In addition, the results (Table 4) indicated that an interaction between time and ratio did not significantly affect the DPPH antioxidant activity of the macadamia, but the interaction between temperature and time or temperature and ratio significantly affected the DPPH antioxidant activity of the macadamia skin.

**Figure 9:**
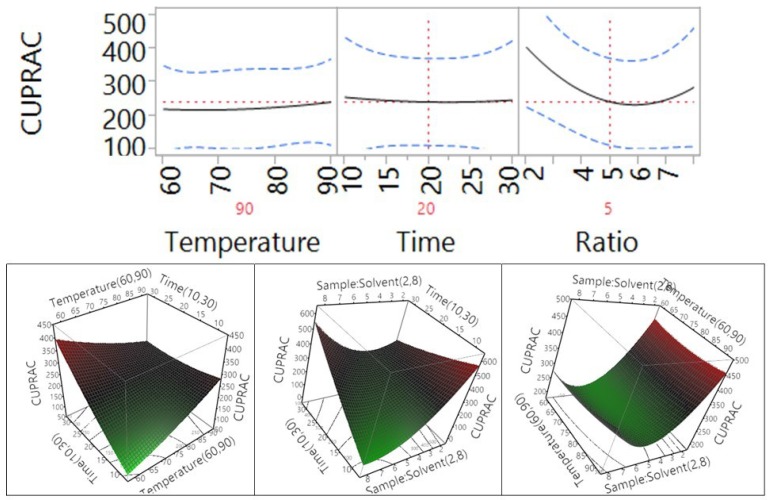
Impact of temperature (60–90 °C), time (10–30 min), and sample-to-solvent ratio (2–8 g/100 mL) on CUPRAC.

The effects of temperature, time, and sample-to-solvent ratio on the cupric ion reducing antioxidant capacity (CUPRAC) of *Macadamia tetraphylla* skin are shown in Table 4 and Figure 9. The results showed that temperature, extraction time, and sample-to-water ratio in the tested range did not significantly affect the CUPRAC antioxidant capacity of the macadamia skin (*p* > 0.05). When the temperature and the time increased from 60 °C to 90 °C and from 10 to 30 min, the CUPRAC antioxidant capacity was not significantly changed; however, the trend of CUPRAC antioxidant capacity decreased when the sample-to-solvent ratio increased from 1 to 8 g/100 mL. There was no significant impact to be observed through the interaction between the temperature and time, temperature and ratio, or time and ratio (*p* > 0.05, Table 4).

**Figure 10:**
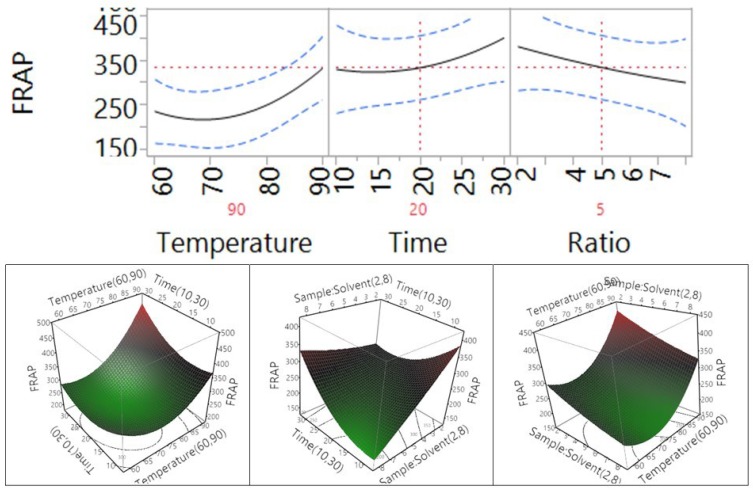
Impact of temperature (60–90 °C), time (10–30 min), and sample-to-solvent ratio (2–8 g/100 mL) on FRAP.

The impact of temperature, time, and sample-to-solvent ratio on the ferric reducing antioxidant power (FRAP) of *Macadamia tetraphylla* skin is shown in Table 4 and Figure 10. Similar to the response of CUPRAC, a temperature, extraction time, and sample-to-water ratio in the tested range did not significantly affect the FRAP antioxidant capacity of the macadamia skin (*p* > 0.05). Interaction between the temperature and time, temperature and ratio, or time and ratio also did not significantly affect the FRAP antioxidant capacity of macadamia skin (*p* > 0.05, Table 4). When the temperature and the time increased from 60 °C to 90 °C and from 10 to 30 min, the trend of FRAP increased; however, the trend decreased when the sample-to-solvent ratio increased from 1 to 8 g/100 mL.

### 3.5. Optimization and Validation of the Models

As seen from the analysis results, the extraction efficiency of TPC, flavonoids, and proanthocyanidins was not significantly affected by the temperature, time, and sample-to-solvent ratio in the tested ranges. However, their extraction efficiency slightly changed when these parameters changed within the ranges. Temperature and time did not significantly affect the antioxidant activity of the macadamia skin, but the ratio significantly affected the DPPH of the extract. Antioxidant activity of the macadamia was varied when the extraction parameters changed. Therefore, it is necessary to identify the optimal conditions at which the optimum values can be obtained for all the chemical and antioxidant properties. Based on the RSM predictive models and expected values, the optimal conditions for total phenolic compounds, flavonoids, proanthocyanidins, and all four different antioxidant assays were selected at temperature = 90 °C, time = 20 min, and sample-to-solvent ratio = 5 g/100 mL.

Validation of the experiments was performed under the previously mentioned optimal conditions to ensure the validity of the models. The predicted and experimental results are shown in Table 5. This showed that there was a strong correlation between the predicted values and the experimental values, amplifying the adequacy of the models and their ability to predict the optimum conditions. Therefore, these conditions can be recommended to be used in future extractions of TPC, flavonoids, proanthocyanidins, and antioxidants from the skin of *Macadamia tetraphylla*. It can also be concluded that RSM is a reliable and effective method of modeling and optimizing extraction conditions. 

**Table 5:** Validation of the predicted values for TPC, flavonoids, proanthocyanidins, and antioxidant potential.

	Values	
	Predicted	Experimental (*n* = 3)
TPC (mg GAE/g)	95.82 ± 50.82 ^a^	86.01 ± 3.45 ^a^
Flavonoids (mg RE/g)	24.03 ± 7.80 ^a^	30.97 ± 3.72 ^a^
Proanthocyanidins (mg GAE/g)	97.31 ± 51.85 ^a^	97.62 ± 5.08 ^a^
ABTS (μM TE/g)	147.34 ± 68.85 ^a^	212.80 ± 14.6133 ^a^
DPPH (μM TE/g)	103.37 ± 39.02 ^a^	130.9111 ± 19.05 ^a^
CUPRAC (μM TE/g)	264.47 ± 216.18 ^a^	476.77 ± 26.46 ^a^
FRAP (μM TE/g)	347.85 ± 141.56 ^a^	558.60 ± 54.35 ^a^

All the values are means ± standard deviations and those in the same row sharing the same superscript letter ^a^ are not significantly different from each other (*p* > 0.05).

It can be seen that the skin of the macadamia is a major source of phenolic compounds, flavonoids, and proanthocyanidins, with an experimental yield of approximately 86.01 mg GAE/g, 30.97 mg RE/g, and 97.62 mg GAE/g of dried sample, respectively. Comparatively, Alasalvar and Shahidi [11] reported that the edible portion of the macadamia contained 1.56 mg GAE/g of total phenolics, while flavonoids and proanthocyanidins were not detected in the edible portion of the macadamia. The identified phenolic content level of the edible kernel represents less than 2% of the phenolic compounds made available by the skin waste, revealing that the skin is very rich in phenolic compounds. In addition, Yang [24] reported that the phenolic and flavonoid content of the macadamia were 4.98 mg/g and 1.38 mg/g, respectively, with the level of proanthocyanidins content undetected. This report did not mention the type of macadamia material (skin, husk, kernel, or the whole) used for analysis, but the level of phenolic content is less than 6% and the flavonoid content is approximately 4.5% of that identified within the skin in the current study under these optimal conditions. 

Alasalvar and Shahidi [11] reported that macadamia nuts have a significant level of total antioxidants ranging from 0.11 to 0.75 mM/100 g. In addition, they also revealed that the FRAP of macadamia nut without skin was approximately 0.42 mM/100 g. In the current study, under the optimal conditions FRAP was found to be 558 μM TE/g or 55.8 mM/100 g, which is approximately 100 times higher. The difference can be explained by the location of the antioxidants mainly in the pellicle (skin) of the nut; less than 10% of the total antioxidant capacity is actually located in the kernel [11]. Therefore, the data showed that macadamia skin is also a potent source of antioxidant. 

## 4. Conclusions

The Box–Behnken design was successfully employed for the optimization of the conventional extraction parameters. RSM was established to be a highly appropriate and reliable tool in evaluating the influence of three individual independent variables (temperature, time, and sample-to-solvent ratio) for the extraction of total phenolic compounds from the skin of *Macadamia tetraphylla.* Within the tested ranges, temperature, time, and sample-to-solvent ratio and their interactions did not significantly affect TPC, flavonoids, and proanthocyanidins. Similarly, these parameters and their interactions did not significantly affect CUPRAC and FRAP; however, the time and the sample-to-solvent ratio significantly affected DPPH antioxidant activity and the ratio significantly affected ABTS antioxidant capacity. The optimal extraction conditions for the extraction of total phenolic compounds, flavonoids, proanthocyanidins, and antioxidant capacity of the skin of *Macadamia tetraphylla* were predicted and validated at a temperature of 90 °C, a time of 20 min , and a sample-to-solvent ratio of 5 g/100 mL.
